# Detecting a familiar person behind the surgical mask: recognition without identification among masked versus sunglasses-covered faces

**DOI:** 10.1186/s41235-022-00440-3

**Published:** 2022-10-04

**Authors:** Brooke N. Carlaw, Andrew M. Huebert, Katherine L. McNeely-White, Matthew G. Rhodes, Anne M. Cleary

**Affiliations:** 1 Department of Psychology, Colorado State University, 1876 Campus Delivery, Fort Collins, CO 80523-1876 USA; 2 Department of Neurology, University of California Davis, Sacramento, CA 95816 USA

**Keywords:** Familiarity-detection, Recognition without identification, Face identification, Butcher-on-the-bus, Surgical masks, Face coverings, Sunglasses, Facial features

## Abstract

Previous research has shown that even when famous people’s identities cannot be discerned from faces that have been filtered with monochromatic noise, these unidentifiable famous faces still tend to receive higher familiarity ratings than similarly filtered non-famous faces. Experiment 1 investigated whether a similar face recognition without identification effect would occur among faces whose identification was hindered through the wearing of a surgical mask. Among a mixture of famous and non-famous faces wearing surgical masks and hoods, participants rated how familiar each person seemed then attempted to identify the person. Though surgical masks significantly impaired identification of the famous faces, unidentified masked famous faces received higher familiarity ratings on average than the non-famous masked faces, suggesting that a sense of familiarity could still occur even when identification was impaired by the mask. Experiment 2 compared faces covered by surgical masks with faces covered by sunglasses. Though sunglasses impaired face identification more than surgical masks, the magnitude of the face recognition without identification effect was the same in both cases. This pattern suggests that holistic face processing is not a requirement for the sense of familiarity with a face, and that different facial feature types can contribute.

## Introduction

The ability to recognize people from faces may be challenging when a face is partially occluded. Recent research spurred by the COVID-19 pandemic suggests that a surgical mask over the mouth and nose of a face impairs individuals’ ability to process that face (Carragher & Hancock, 2020; Estudillo et al., 2021; Freud et al., 2020). In the present study, we investigate whether people can sense familiarity with a surgically masked face when the masked person cannot be consciously identified, in a phenomenon that might be akin to recognition without identification (RWI) of noise-masked faces (e.g., Cleary et al., 2013), or whether surgical masks prevent the type of familiarity-detection with a face that would allow for such RWI to occur. We additionally compare the effects of surgical masks vs. sunglasses on face RWI.

### Impairments to face processing from surgical masks

Research occurring since the start of the COVID-19 pandemic suggests that surgical masks create substantial impairments in various aspects of face processing (Carragher & Hancock, 2020; Estudillo et al., 2021; Freud et al., 2020; Stajduhar et al., 2022). For example, Freud et al. (2020) administered the Cambridge Face Memory Test (CFMT; Duchaine & Nakayama, 2006), a standardized measure of face recognition that asks participants to first learn a set of novel faces and then later choose a studied face from among three alternatives. Overlaying faces in the CFMT with surgical masks significantly impaired performance relative to the standard version involving unmasked faces. Carragher and Hancock (2020) used a matching task that required participants to determine whether two faces presented side-by-side represented the same or different individuals. Relative to a control condition of faces presented without masks, participants’ matching accuracy was significantly poorer when one or both faces were masked.

### Recognition without identification (RWI)

Familiarity-detection is an aspect of face processing that has yet to be investigated in the context of surgical masks. Familiarity-detection with faces is best illustrated by the now famous butcher-on-the-bus example (MacLeod, 2020; Mandler, 1980), whereby a person onboard one’s bus might seem oddly familiar while the experiencer struggles to identify why (i.e., that the familiar-seeming person is the local butcher). The method that we use to study this aspect of face processing in the present study is a variant of the RWI paradigm.

RWI is the finding that, among stimuli that have been presented in such a way as to impede identification, a sense of recognition is often still present. Most forms of RWI have been shown using list-learning paradigms, whereby among unidentified stimuli (e.g., a word fragment that cannot be identified, such as R_ I_ _R _P), participants discriminate unidentified stimuli that came from a stimulus presented on an earlier study list (e.g., from the study list word RAINDROP) from stimuli that did not, such as by giving higher recognition or familiarity ratings to the former (e.g., Cleary, 2002; Cleary & Greene, 2000, 2001, 2004; 2005; Cleary et al., 2004, 2007, 2010; Kostic & Cleary, 2009; Langley et al., 2008; McNeely-White & Cleary, 2019; McNeely-White et al., 2021; Morris et al., 2008; Peynircioğlu, 1990).

Most germane to the present study, RWI has also been examined in more life-like situations of familiar-novel discrimination rather than just situations of studied-unstudied discrimination, including familiar-novel discrimination among unidentified face stimuli (Cleary et al., 2013). Famous and non-famous faces were embedded within a visual noise mask created by applying a Gaussian monochromatic noise filter that made the entire face appear hazy and blurred. RWI was shown by a pattern of higher familiarity ratings for unidentified famous faces (i.e., known faces that could not be identified through the mask) than non-famous noise-masked faces. This variant of the RWI phenomenon may better approximate the type of RWI that occurs in the real world, where the task is to recognize whether something is known vs. novel (rather than recently studied). In the present study, we used this approach to examine whether higher familiarity ratings would be given to famous faces whose identification was impeded by a surgical mask than to non-famous surgically masked faces.

### The current study

In the present study, we aimed to determine whether participants would be able to discriminate between unidentifiable famous vs. non-famous faces when a surgical mask impedes identification. Given prior work suggesting detrimental effects of a surgical mask on various aspects of face processing (Carragher & Hancock, 2020; Freud et al., 2020), we anticipated that the mask would hinder identification of famous faces. Our primary question was whether familiarity detection would occur despite the hindered identification.

Determining whether familiarity-detection with unidentifiable faces can occur when major features of the face (e.g., the nose and mouth) are occluded will inform theory regarding the familiarity-detection mechanism. Other forms of familiarity-detection examined using the RWI paradigm may reflect a feature matching process whereby features present in a cue stimulus (e.g., a song’s rhythm) are matched with separable features stored in memory (e.g., recently heard rhythms) to produce a familiarity signal of varying intensity depending on the degree of global feature match (e.g., McNeely-White et al., 2021). If this type of global feature matching mechanism (see Clark & Gronlund, 1996, for a review) can also occur with faces, then it is reasonable to hypothesize that occluding major facial features like the nose and mouth (as occurs with a surgical mask) could still enable familiarity-detection from the remaining features available (in this case, eye and forehead information) to be matched with features stored in memory for known faces.

However, there is reason to hypothesize that faces are less likely to be decomposed into constituent features than other types of stimuli and are instead more likely to be processed holistically (Bruce & Young, 2012; Maurer et al., 2002; Meltzer & Bartlett, 2019; Tanaka & Farah, 1993; Tanaka & Simonyi, 2016), which may be a reason why surgical masks disrupt so many different aspects of face processing (Carragher & Hancock, 2020; Estudillo et al., 2021; Freud et al., 2020; Stajduhar et al., 2022). If so, then familiarity-detection during face identification failure might be impeded by occluding the nose and mouth with a surgical mask, as evidence suggests that surgical masks disrupt holistic processing of faces (Stajduhar et al., 2022). In short, finding RWI with the masked faces would suggest that isolated facial features (in this case, eye information) can produce the effect, whereas a failure to find RWI might suggest that holistic processing of faces is required for face RWI to occur, a process disrupted by surgical masks.

Searching for an RWI effect among faces covered by surgical masks enabled us to also measure the level of face identification impairment caused by surgical masks using a completely different approach than applied previously. Specifically, by examining the level of famous face identification among masked celebrities whose unmasked faces were later identifiable by the participant, we could determine the within-subject decrement to famous face identification performance caused by a surgical mask covering. Thus, the present study enabled us to address two primary questions: (1) Is RWI shown among faces whose identification was impeded by a surgical mask, and (2) To what degree is identification of a known person impaired by the surgical mask covering?

## Experiment 1

### Method

#### Participants

Fifty-four participants were recruited from the online research platform Prolific in August 2021. Sample size was determined using G*Power and the medium effect size (Cohen’s *d* = 0.52) for noise-masked face RWI found by Cleary et al. (2013). A power of 95% with an alpha of 0.05 for a medium effect size (0.50) would require a sample size of 54. Due to the likely cultural specificity of the celebrity images used in the present study, we limited participant recruitment to people whose current country of residence is the USA or Canada, and whose first language is English. We also limited recruitment to people without impaired vision. Participants received compensation ($6.40/half-hour) for their participation, which took approximately 30 min. No demographic data were collected; however, the authors received notice from Prolific that during the time window in which the data were collected, there were more female-identifying participants in the pool. Therefore, although we did not collect gender identity information, our sample likely contained more female-identifying participants than other gender identities.

#### Materials

The experiment was implemented online using Qualtrics. The stimuli were 30 images of celebrities’ faces and 30 images of non-famous actors’ and actresses’ faces that had all been selected from the Internet Movie Database (IMDb) during the period between 2006 and 2016 and saved as JPEG files. All images came from a pool used in prior research studies (Cleary, 2019; Cleary & Specker, 2007; Cleary et al., 2013), and for which the celebrities had been chosen for being well-known in movies, TV shows, and/or the music industry. From among these, we chose 30 celebrities who would be considered currently famous, A-list celebrities (e.g., Blake Shelton, Elijah Wood, and Kristen Stewart). From among the non-famous faces in the pool, we selected 30 B-list actors and actresses whose headshots came from the IMDb during the period between 2006–2007 and were selected for comparable quality to those of the famous A-list celebrities while being of people highly unlikely to be known to our participants. All images are available at: https://osf.io/28s6n/.

**Fig. 1:**
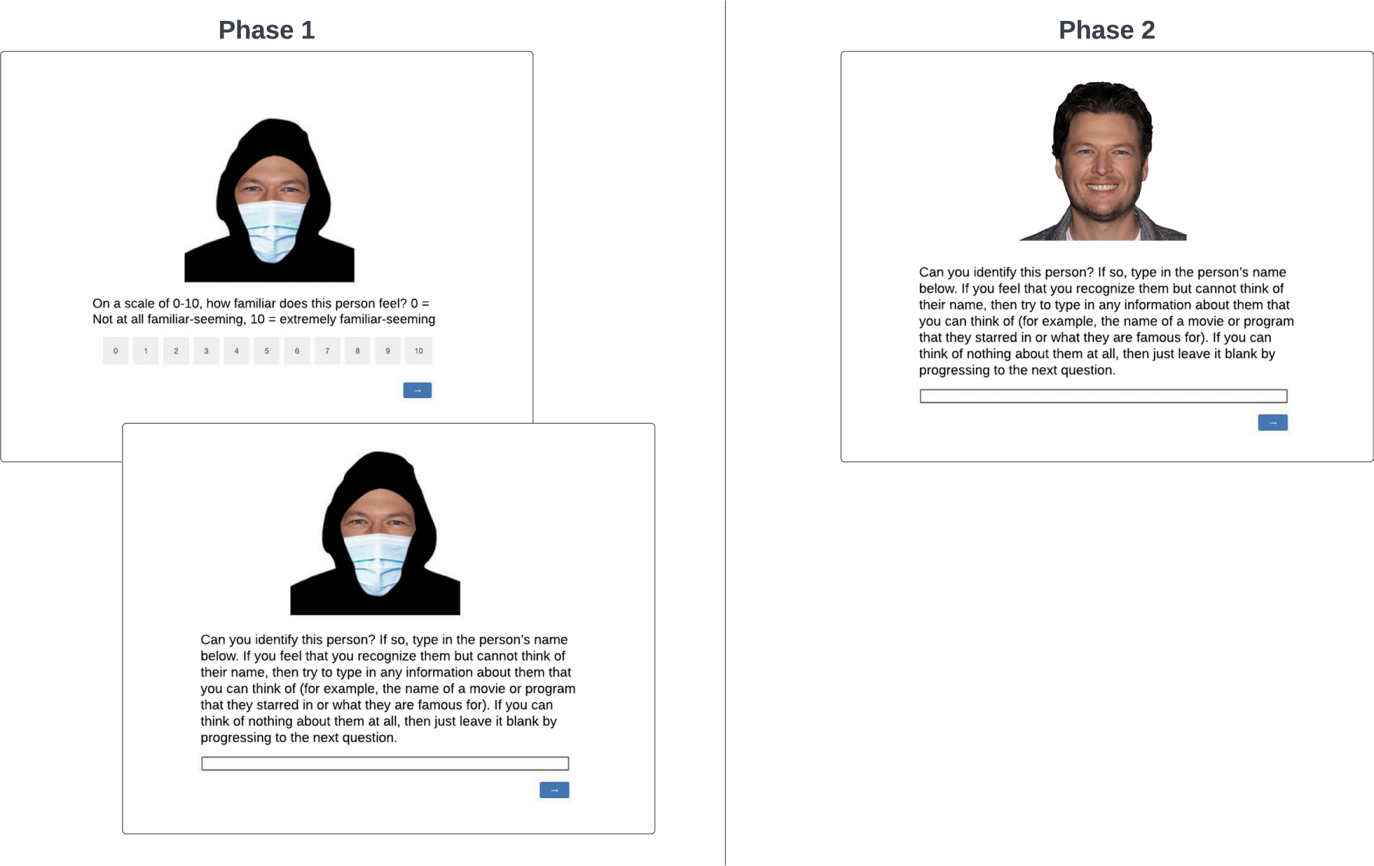
Example of the procedure. The celebrity face Blake Shelton is depicted along with the prompts shown during Phase 1 and then unmasked and unhooded in Phase 2. Original celebrity image from DFree / Shutterstock.com

Of the 30 celebrity faces, 14 were female and 16 were male, and likewise for the 30 non-famous faces. All images were resized to 175 × 175 pixels and the background changed to white using Adobe Photoshop. Adobe Photoshop was also used to cover each of the 60 faces with a blue surgical face mask over the nose and mouth area and the hair with a black hood for Phase 1 of the experiment (see Fig. 1 for an example). The blue surgical face mask and black hood images that were overlaid over the headshots were selected from SearchPng (searchpng.com) and PNGIX (pngix.com) respectively and saved as PNG files. For Phase 2, the 30 celebrity faces were presented one at a time without the surgical face mask and hood (see Fig. 1) to assess whether the celebrities appearing in Phase 1 would have been identifiable if unmasked and unhooded.

#### Procedure

The procedure is depicted in Fig. 1. After completing the consent form, participants were presented with the experiment instructions (available at: https://osf.io/28s6n/). Then, the Phase 1 trials began, which consisted of 60 masked, hooded faces, 30 of which were of famous celebrities and 30 of which were of non-famous actors’ headshots, presented in a random order. The first masked, hooded face appeared in the center of the screen (left side of Fig. 1) with a forced-choice prompt for a familiarity rating (see Fig. 1). Once a rating was selected, the next prompt appeared while the face image remained on the screen and asked for an identification response (Fig. 1). Each of the Phase 1 trials were self-paced.

Once the participant completed each of the 60 masked, hooded face trials from Phase 1, Phase 2 began (see https://osf.io/28s6n/ for instructions). As Phase 2 served to determine which of the masked, hooded famous faces from Phase 1 would have been identifiable to the participant if unmasked and unhooded, all 30 of the celebrity faces from Phase 1 were presented unmasked and unhooded in a random order. For each, participants were prompted to provide an identification response as shown in the right side of Fig. 1. At the end of Phase 2, the participant was provided with a brief explanation of the experiment and directed to the screen for receiving compensation for participating.

## Results

Every trial containing a famous (celebrity) face from Phase 1 (masked and hooded trials) and Phase 2 (unmasked and unhooded trials) was manually classified as either an identified, partially identified, unidentified or misidentified trial. Trials were classified as identified when the celebrity’s first and last name was typed (e.g., “Blake Shelton”), even if misspelled (e.g., “Blake Sheltan”). Trials were classified as partially identified when only the first or last name was indicated (e.g., “Blake”), or accurate specific information about the celebrity was typed (e.g., “Country music singer judge on ‘*The Voice*’). Trials were classified as unidentified when no identification attempt was made (the prompts were left blank), when incorrect information was typed, or when the information typed was vague enough to be applicable to many faces in the pool (e.g., “Actor” or “Singer”). Occasionally, a participant provided an identical incorrect identification in both phases, indicating that the participant would have identified the face the same incorrect way when masked as when unmasked (e.g., a subject typed “Robert De Niro” as their identification attempt in Phase 1 [masked] and Phase 2 [unmasked] for the celebrity, Bryan Cranston). Such instances were labeled as misidentified so as not to be included in the computation of celebrity identification rates. As the primary focus in the present study is on unidentified faces, for our purposes, trials in which either full or partial identification took place are considered instances of successful face identification.^1^

Our primary focus is on familiarity ratings given during instances in which the surgical mask impaired famous face identification for someone who would otherwise have been an identifiable person to the participant. Accordingly, it is important to consider how many trials contributed to the mean familiarity ratings in these instances. To ensure that a reasonable number of trials contributed to the mean, we used a criterion of at least three trials contributing to the mean for inclusion. Only two of the 54 participants in question had fewer than three trials to contribute to the famous condition mean familiarity rating (noted in Footnote 1); the average number of trials contributing to the means for the remaining 52 was 10.75 (*SD* = 4.83) with a range of 3–21.

### Full and partial identification frequencies

In Phase 2, out of the 30 unmasked, unhooded celebrity faces (depicted in the right-hand panel of Fig. 1), participants fully identified an average of 11.79 (*SD *= 6.16) faces, and partially identified an average of 6.15 (*SD* = 3.49) faces. In Phase 1, out of the 30 masked, hooded celebrity faces (depicted in the left-hand panel of Fig. 1), participants fully identified an average of 4.77 (*SD* = 4.14) and partially identified an average of 2.54 (*SD* = 2.57).

### Decrement to famous face identification

We first examined the extent to which the combination of the surgical mask and hood hindered identification of what would otherwise have been identifiable famous faces for a given participant. To determine if a face was known to an individual participant, we examined the participant’s ability to identify that person unmasked and unhooded. Specifically, we determined for each participant which of the unmasked famous faces from Phase 2 were either fully or partially identified; based on this, we then examined how many of those celebrities were identifiable (either fully or partially) from among the masked faces depicted in Phase 1. Any decrement to identifiability of a celebrity from Phase 2 to Phase 1 provides an estimate of the degree to which the loss of the nose, mouth and hair information impeded face identification. If 100% of the faces that were identified in Phase 2 were also identified when masked and hooded in Phase 1, it would mean that masking and hooding caused no decrement.^2^ On average, among those unmasked celebrity faces that were identifiable (either fully or partially) to a participant in Phase 2, only 0.38 (*SD* = 0.23) of them could be identified (fully or partially) when masked and hooded in Phase 1. A one-sample *t* test comparing the sample against 1.0 (perfect identification) revealed that this probability of correct identification of masked faces (0.38) was significantly lower than 1.0, *t*(51) = -19.70, *SE* = 0.03, *p* < 0.001, *BF*_*10*_ = 2.071 × 10^22^. All Bayes Factors were calculated using JASP and the JZS prior. Classifications of evidence strength adhered to the recommendations of Wagenmakers (2007). Being limited to only a person’s eye and forehead information substantially limited overall face identification ability.

### Recognition without identification (RWI)

Our primary interest was whether RWI would occur based on the available eye and forehead information present among the unidentified faces whose identification was hindered. Indeed, among unidentified masked, hooded celebrity faces that would have been identifiable if unmasked and unhooded, familiarity ratings were higher (*M* = 2.60, *SD *= 1.63) than among masked, hooded non-famous faces (*M* = 1.65, *SD* = 0.97), *t*(51) = 6.68, *SE* = 0.14, *p* < 0.001, *d* = 0.58, *BF*_*10*_ = 7.44 × 10^5^.^3^ In short, participants detected increased familiarity with famous faces whose identification was prevented by the surgical mask and hood manipulation relative to comparable non-famous faces wearing the hood and surgical mask. Thus, the eye and forehead information available among these faces could still be used to detect familiarity with the faces.

## Experiment 2

Experiment 1’s results suggest that although surgical face masks significantly impair face identification, familiarity with a known face is still detectable even among unidentified faces. This points toward the idea that certain facial features can allow for familiarity-detection with a face (Abudarham et al., 2019). In particular, a face’s eye information, in the absence of nose and mouth information, can elicit familiarity-detection. Some research suggests that eye information may have greater importance to global face processing than other facial features (e.g., Diego-Mas et al., 2020; Fox & Damjanovic, 2006). Along these lines, some research suggests that sunglasses may be as impairing to many aspects of face processing as surgical masks (Bennetts et al., 2022; Noyes et al., 2021). To investigate whether eye information is special, or if other facial features (i.e., nose and mouth information) can similarly produce face RWI, Experiment 2 examined how familiarity-detection from unidentified surgically masked faces compares with familiarity-detection from unidentified sunglasses-covered faces.

### Method

#### Participants

Based on Experiment 1’s sample size estimate and a stop date, 112 students from Colorado State University participated in-person in exchange for credit toward a course. They were alternatingly assigned to either the masks (55 total) or sunglasses (57 total) condition upon arrival to the laboratory.

#### Materials

**Fig. 2:**
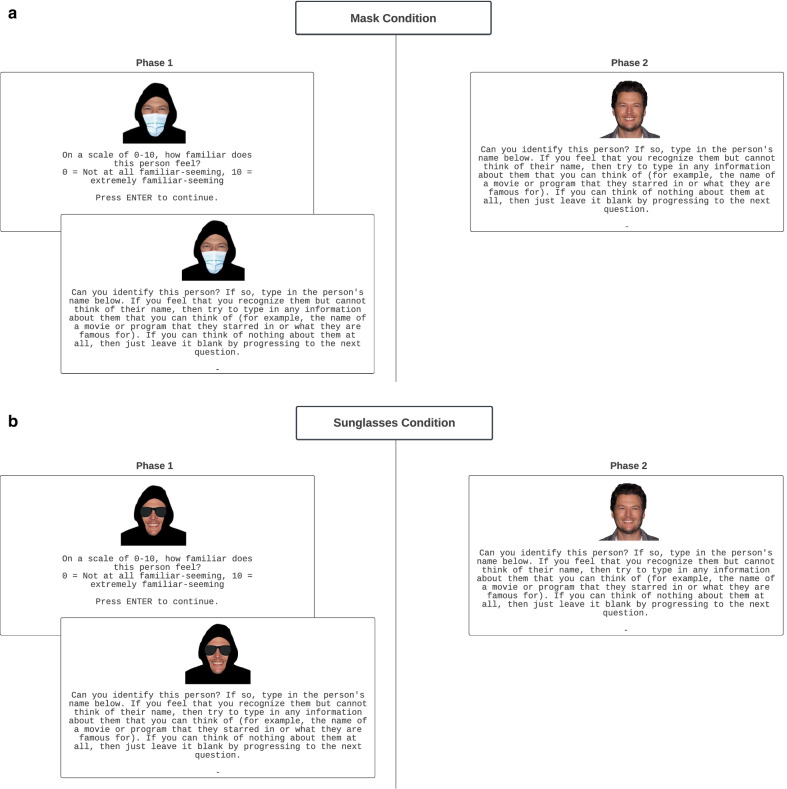
**a** Example of the procedure for the surgical face mask condition. The celebrity face Blake Shelton is depicted along with the prompts shown during Phase 1 and then unmasked and unhooded in Phase 2. Original celebrity image from DFree/Shutterstock.com. **b** Example of the procedure for the sunglasses condition. The celebrity face Blake Shelton is depicted along with the prompts shown during Phase 1 and then unmasked and unhooded in Phase 2. Original celebrity image from DFree / Shutterstock.com

The experiment was implemented in individual computer booths using E-Prime. The stimuli were the same as those used in Experiment 1 with the exception that the image size was changed from 175 × 175 pixels to 300 × 300 pixels to display properly in E-Prime. For the sunglasses condition, rather than a blue surgical mask covering the nose and mouth area, each of the 60 faces was covered with an image of sunglasses over the eye area for Phase 1 (see Fig. 2b for an example). The sunglasses image was selected from Klipartz (klipartz.com) and saved as a PNG file.

#### Procedure

The procedure is depicted in Fig. 2a, b and was identical to that used in Experiment 1 with the following exceptions. The specific prompts differed slightly between the Qualtrics version used in Experiment 1 and the E-Prime version used in Experiment 2. Notably, whereas participants mouse-clicked on a number from the fully depicted familiarity ratings scale in Experiment 1, in Experiment 2 participants needed to type an integer between 0 and 10 before pressing Enter to continue.

Due to a programming error, in Phase 2 of the sunglasses condition, the 30 images of the non-sunglasses-covered celebrity faces from Phase 1 were not randomized; however, because this phase was simply meant to index which celebrity faces were known to the participant vs. which were not, it should not compromise the findings. Moreover, an independent samples *t* test performed on the total full and partial Phase 2 identification rates between the masks (*M* = 14.04, *SD* = 5.83) and sunglasses conditions (*M* = 13.85, *SD* = 5.60) revealed no significant difference, *t*(101) = 0.17, *SE* = 1.13, *p* = 0.86, *BF*_*01*_ = 4.74.

## Results

Trial classification was performed manually as in Experiment 1. None of the Colorado State University participants in Experiment 2 successfully identified or partially identified Ali Larter when unmasked in Phase 2. Therefore, this stimulus was removed from the pool of data under consideration (as this pattern indicates that Ali Larter was unknown to this participant population). Note that because the focus is on familiarity ratings given to unidentified occluded faces that would have been at least partially identifiable if unoccluded, this does not impact the findings, as there would not have been any such instances to include for this stimulus anyway.

Two participants were lost from the masks condition (one for not identifying any of the unmasked celebrities, and one for identifying all masked celebrities that were identifiable later on when unmasked), and two were lost from the sunglasses condition due to not completing the experiment, leaving 53 in the masks condition and 55 in the sunglasses condition. Two of the 53 participants in the masks condition and three of the 55 in the sunglasses condition did not meet the minimum of three trials contributing to the familiarity rating mean in the famous condition, leaving 51 in the masks condition and 52 in the sunglasses condition. The average number of trials contributing to the familiarity rating mean among the included participants was 9.37 (*SD* = 3.44) with a range of 3–17 in the masks condition and 11.33 (*SD* = 4.27) with a range of 3–22 in the sunglasses condition.

### Full and partial identification frequencies

In Phase 2 of the mask condition, out of the 29 unmasked, unhooded celebrity faces (depicted in the upper right-hand panel of Fig. 2a), participants fully identified an average of 6.37 (*SD* = 4.28) of them, and partially identified an average of 7.67 (*SD* = 3.43) of them. In Phase 1, out of the 29 masked, hooded celebrity faces (depicted in the upper left-hand panel of Fig. 2a), participants fully identified an average of 2.71 (*SD* = 2.97) and partially identified an average of 2.10 (*SD *= 2.18).

In Phase 2 of the sunglasses condition (depicted in the lower-right-hand panel of Fig. 2b), participants fully identified an average of 5.21 (*SD* = 3.69) and partially identified an average of 8.47 (*SD* = 4.03) of the celebrity faces. In Phase 1 (depicted in the lower left-hand panel of Fig. 2b), participants fully identified an average of 1.17 (*SD* = 1.35) and partially identified an average of 1.52 (*SD* = 1.59) of the celebrity faces.

### Decrement to famous face identification

As in Experiment 1, we determined for each participant what proportion of would-be fully or partially identifiable celebrities (in Phase 2) were fully or partially identifiable from among the occluded faces depicted in Phase 1. If facial occlusion had no effect, the same faces that were at least partially identifiable in Phase 2 would also have been at least partially identifiable in Phase 1. One-sample *t* tests revealed that the proportion of would-be identifiable faces that were identified when occluded was significantly lower than 1.0 in both the mask condition (*M* = 0.28, *SD* = 0.21), *t*(50) = − 24.34, *SE* = 0.03, *p* < 0.001, *BF*_*10*_ = 1.26 × 10^26^, and the sunglasses condition (*M* = 0.16, *SD* = 0.12), *t*(51) = -51.39, *SE* = 0.02, *p* < 0.001, *BF*_*10*_ = 9.45 × 10^41^. An independent samples *t* test revealed that sunglasses were significantly more impairing to face identification than surgical masks, *t*(78.05) = 3.54, *SE* = 0.03, *p* < 0.001, *d* = 0.70, *BF*_*10*_ = 48.30.^4^

### Recognition without identification (RWI)

Our primary interest was in whether face RWI would depend on the type of occlusion impeding identification (masks vs. sunglasses). A 2 (Famousness Status: famous, non-famous) × 2 (Occlusion-type: mask, sunglasses) mixed-factor ANOVA performed on familiarity ratings given to famous faces unidentified in Phase 1 but at least partially identifiable in Phase 2 and to non-famous faces revealed that, overall, familiarity ratings were higher for famous than non-famous faces, *F*(1, 101) = 93.68, *MSE* = 0.53, *p* < 0.001, *η*_*p*_^2^ = 0.48, *BF*_*10*_ = 1.15 × 10^13^, but that there was no difference based on the type of occlusion, *F*(1, 101) = 2.97, *MSE* = 3.65, *p* = 0.09, *η*_*p*_^2^ = 0.03, *BF*_*01*_ = 1.08. Importantly, there was also no interaction, *F*(1, 101) = 0.03, *p* = 0.85, *η*_*p*_^2^ = 0.00, *BF*_*01*_ = 4.71. Thus, RWI occurred to the same extent for faces occluded by sunglasses, *t*(51) = 7.92, *SE* = 0.13, *p* < 0.001, *d* = 0.66, *BF*_*10*_ = 7.90 × 10^4^, as for faces occluded by masks, *t*(50) = 6.05, *SE* = 0.16, *p* < 0.001, *d* = 0.63, *BF*_*10*_ = 5.37 × 10^7^.

## General discussion

### Overview

The present study examined whether face RWI would occur among unidentified faces whose identification was hindered by the presence of a surgical mask and hood (Experiment 1) and compared RWI among faces whose identification was hindered by a surgical mask vs. by sunglasses (Experiment 2). The presence of a surgical mask and hood markedly impeded identification of a known, famous face. In Experiment 1, only 38% of famous faces that were identifiable when unmasked and unhooded were identified by participants when masked and hooded. Among the roughly 62% of known faces whose identification was prevented by the presence of the mask and hood in Experiment 1, RWI was shown: Significantly higher familiarity ratings were given to unidentified masked famous faces that were identifiable without the mask and hood than to masked non-famous faces.

**Fig. 3:**
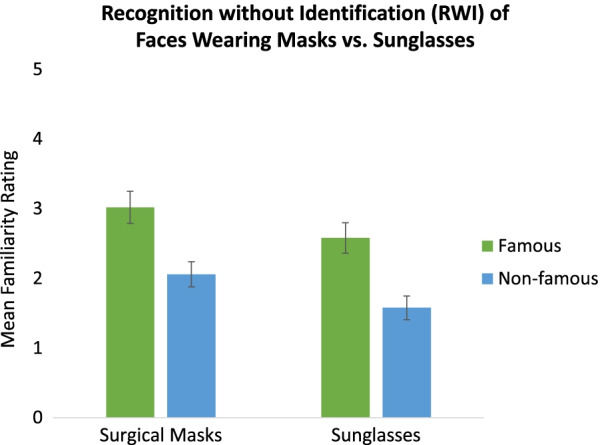
The Face Recognition without Identification (RWI) effect—discriminability via higher familiarity ratings for unidentified occluded famous faces than occluded non-famous faces—was comparable in magnitude when faces were unidentified from surgical masks versus from sunglasses. There was no significant main effect of occlusion-type (masks vs sunglasses)

Experiment 2 compared face RWI when facial feature occlusion occurred via surgical masks (covering the nose and mouth) or via sunglasses (covering the eyes and eyebrows). Sunglasses in the presence of a hood were significantly more impairing to identification of known, famous faces than surgical masks in the presence of a hood. Only 16% of famous faces that were identifiable when unmasked and unhooded were identified by participants when wearing sunglasses and a hood, whereas 28% were identified when they were instead wearing a surgical mask and a hood. Despite the greater degree of face identification impairment caused by sunglasses as opposed to by surgical masks, the RWI effect was present to the same degree regardless of whether identification was impaired by sunglasses or masks (see Fig. 3).

### Future directions

An intact familiarity-detection mechanism in the face of identification impairment may reflect important mechanisms used by the larger cognitive system. For instance, familiarity-detection might prompt the initial memory search for information potentially relevant to the situation at hand (Huebert et al., 2022). Along these lines, some have suggested that the feeling of familiarity drives people to attempt to conjure up any potentially related information to the current situation that will come to mind, even if it means self-generating information that is not actually related to the situation at hand (Moulin, 2013, 2018). If so, then the ability to detect familiarity with a face might serve the useful purpose of prompting a person to attempt to generate potentially relevant information to determine the identity of the unidentified person.

Elements of our data provide some evidence that participants may have made greater efforts to actively search memory for potentially relevant information in the face of a sense of familiarity with a yet unidentified face. There are two ways a trial could be classified as unidentified: (1) leaving the identification response prompt blank (an omission error) or (2) typing incorrect information into the prompt (a commission error). In Experiment 1, participants exhibited a higher probability of making commission errors (as opposed to omission errors) among unidentified masked would-be identifiable faces (*M* = 0.27, *SD* = 0.21) than among masked non-famous faces (M = 0.1*5*, *SD* = 0.11), *t*(51) = 5.79, *SE *= 0.02, *p* < 0.001, *d* = 0.62, *BF*_*10*_ = 3.45 × 10^4^ (see footnote 3). A similar pattern was shown in Experiment 2.^5^ Participants typed more commission errors in response to unidentified but known surgical mask-covered famous faces (*M* = 0.22, *SD* = 0.22) than to surgical mask-covered non-famous faces (*M* = 0.14, *SD* = 0.14), *t*(50) = 3.68, *SE* = 0.02, *p* < 0.001, *d* = 0.39, *BF*_*10*_ = 46.39 (see footnote 3). They also typed more commission errors in response to unidentified but known sunglasses-covered famous faces (*M* = 0.20, *SD* = 0.20) than to sunglasses-covered non-famous faces (*M* = 0.10, *SD* = 0.10), *t*(51) = 4.24, *SE* = 0.02, *p* < 0.001, *d* = 0.55, *BF*_*10*_ = 238.44 (see footnote 3).

Although these exploratory analyses of commission error patterns are correlational in nature, they do raise the interesting possibility that participants make more commission errors for unidentified but known (familiar) occluded faces than for unknown (unfamiliar) occluded faces because familiarity-detection with a face prompts a memory search for potentially relevant information. In this way, this pattern may be revealing an important and more general aspect of the memory system and its operation. If familiarity-detection prompts memory search, then it may be a process that can be capitalized on for training purposes for helping people to improve their face recognition abilities in situations where faces are occluded or hard to identify. Future research should continue to investigate this possibility, such as by examining the time course and temporal dynamics of people’s memory judgments in relation to the production of articulable information.

### Limitations

The present study has important limitations to also consider in future research. One limitation of the present study is that we did not record demographic information from our participants. In the future, it will be important to collect demographic information from the participants as well as to diversify the pool of stimuli under examination so that patterns such as cross-race bias effects can be explored. For example, is the face RWI effect reported here affected by the cross-race bias (e.g., Trawiński et al., 2021)?

Another potential limitation concerns the difficulty our participants had identifying the famous faces even when they were presented unoccluded in Phase 2. As the famous faces used in the present study came from an older set, they were likely not as known to our participants as an updated set would be. Accordingly, an interesting question for future research using an updated or more identifiable stimulus set is whether surgical masks and sunglasses would have the same impairing effect on identification of known faces when unoccluded face identification is more likely. It will likewise be important to examine face RWI in the presence or absence of hoods.

The non-celebrity faces were also from a relatively old set (from over a decade ago). As these faces were of amateur actors from some time ago, it is highly unlikely that our participants would have known or been familiar with any of them (in fact, as we note above, identification rates were not high even for the celebrities used in the present study). Our laboratory did not retain names corresponding to the non-famous face images. However, for anyone interested in further investigating specifically what participants typed for these faces, the raw data can be found on the OSF. Of note, not only did participants type information less often for non-famous faces (see the commission vs. omission data described above), but they tended to guess famous people’s names when they did type something.

Finally, in the present experiments, the status of a face as famous vs. non-famous cannot be counterbalanced, raising the possibility of item-based effects. Although our famous and non-famous face stimulus sets were intended to be comparable in factors such as the nature and quality of the headshot, the attractiveness of the person, etc., it remains possible that the sets differ on an important dimension. To study the type of face RWI explored in the present study in a way that allows for counterbalancing of the stimulus faces across conditions, future research could explore ways of training participants on novel faces so that the comparison is between trained and untrained faces, rather than between already famous and non-famous faces.

## Conclusions

The significant impairment to face identification caused by surgical masks is perhaps not surprising in light of recent research on the various ways in which surgical masks impair face processing (e.g., Carragher & Hancock, 2020; Estudillo et al., 2021; Freud et al., 2020). However, the present study extends these findings by showing dramatic impairment to identification of known faces that would have been identifiable to the participants if not occluded by a surgical mask, and by showing even more dramatic impairment to identification of known faces when sunglasses instead of surgical masks are occluding parts of the face. Our results converge on a growing body of evidence that the everyday task of trying to identify and interact with familiar individuals who are wearing surgical masks is likely significantly impeded, particularly when a hair covering like a hood is also present. However, our results suggest that surgical masks are still not as impairing as when familiar individuals are wearing sunglasses and a hood with no mask.

The most novel aspect of our study concerns the face RWI effect. Our findings suggest that even when identification is significantly impaired by a facial feature-blocking occlusion like a surgical mask or sunglasses, familiarity-detection with a known face can still occur based on the facial features remaining available. Such data have significant implications for theory regarding how familiarity-detection occurs with faces. Although several theoretical approaches to face processing emphasize a role of holistic representations (Bruce & Young, 2012; Manley et al., 2019; Maurer et al., 2002; Meltzer & Bartlett, 2019; Tanaka & Farah, 1993; Tanaka & Simonyi, 2016), global matching models of familiarity signal computation emphasize a decomposition of stimuli into constituent features (Clark & Gronlund, 1996; McNeely-White et al., 2021). From a global matching perspective, familiarity-detection should occur for partially occluded faces via a matching of the available features present in the occluded face with facial features stored in memory to yield a higher familiarity signal for features coming from known (famous) faces than for features coming from unknown (new) faces. The present findings suggest that eye information and nose and mouth information are types of facial features that may be able to participate in a global feature matching process to allow for familiarity-detection with an unidentified face. Future research should further examine the extent to which facial familiarity-detection involves feature matching.

## Data Availability

The data and materials are available on the OSF at the following link: https://osf.io/28s6n/.

## Abbreviation

RWI: Recognition without identification

## Footnotes

1 One participant was lost from the reported analyses after failing to successfully fully or partially identify any of the celebrities in either phase, and another was lost for not exhibiting any failure to identify (partially or fully) masked celebrities that were at least partially identifiable in Phase 2 (in short, this person had no decrement to identification ability from the surgical masks).

2 This occurred for only one participant, and as a result, that person had no familiarity rating data in the condition of interest (see Footnote 1).

3 The effect size reported is Cohen’s *d* for repeated-measures (drm; see Lakens, 2013).

4 Levene’s test showed unequal variances (see Levene, 1960).

5 In Experiment 2, a 2 Famousness Status (famous, non-famous) × 2 Occlusion-type (mask, sunglasses) mixed ANOVA performed on the probability of a commission error for faces unidentified in Phase 1 but at least partially identifiable in Phase 2 revealed a significant main effect of Famousness Status, *F*(1, 101) = 31.37, *MSE* = .01, *p* < .001, *η*_*p*_^2^ = .24 (whereby higher commission errors were given to unidentified famous than to non-famous faces), no main effect of Occlusion-type, *F* < 1.0, and no interaction, *F* < 1.0.
